# Black Hole in My Child’s Mouth: An Unusual Case of a Foreign Body Mimicking a Palatal Lesion in a Healthy Infant

**DOI:** 10.7759/cureus.96133

**Published:** 2025-11-05

**Authors:** Noor Sadiq Syed, Naveen Nagalla

**Affiliations:** 1 Emergency Medicine, Mid Yorkshire Teaching NHS Trust, Wakefield, GBR

**Keywords:** emergency medicine and trauma, foreign bodies in emergency medicine, general paediatrics, maxillo facial surgery, otorhinology

## Abstract

Foreign bodies in the oral cavity of children can pose significant diagnostic challenges, particularly when they are asymptomatic and mimic serious pathological lesions. We report an unusual and deceptive case involving a 16-month-old infant who presented with what appeared to be a suspicious black lesion firmly adhered to the hard palate. Upon further investigation, the lesion was identified as a plastic emblem dislodged from a car key fob, firmly stuck due to adhesive residue. This case underscores the critical importance of thorough history-taking, detailed and careful oral examination, and the need to consider foreign bodies in the differential diagnosis of oral lesions. Early recognition and appropriate management can prevent unnecessary investigations, avoid misdiagnosis, and reduce caregiver anxiety.

## Introduction

Foreign body ingestion and impaction are among the most frequent emergencies encountered in pediatric practice, particularly in children under five years of age, owing to their exploratory oral behavior, developmental curiosity, and limited awareness of potential hazards [1]. While most ingested foreign bodies pass harmlessly through the gastrointestinal tract, a small subset becomes lodged within the upper aerodigestive or oral cavities, creating both diagnostic and management challenges. When such foreign bodies present atypically, especially when asymptomatic or mimicking true mucosal pathology, they can lead to misinterpretation, unnecessary imaging, or even invasive intervention [2].

The oral cavity, despite being easily accessible to inspection, can harbor deceptively adherent or radiolucent objects that resemble ulcerative or necrotic lesions. Plastic, organic, or adhesive materials in particular may attach firmly to the mucosal surface and appear as dark or irregular “growths,” prompting concern for infection, vascular malformation, or malignancy. Because many of these materials are radiolucent, conventional radiographs often fail to identify them, further compounding diagnostic uncertainty [3]. In such scenarios, meticulous visual inspection, gentle palpation, and a thorough environmental history are vital in differentiating a benign foreign body from true pathology.

Although foreign body impaction in the esophagus and oropharynx is well-documented, reports describing adherence of small objects to the hard palate are exceedingly rare. Previous case reports have described palatal stickers, shells, or fragments of plastic mimicking mucosal tumors or necrotic patches, sometimes persisting for weeks or months before recognition [2,3]. These uncommon presentations highlight the importance of considering foreign bodies within the differential diagnosis of any unusual oral lesion, particularly in the mouthing age group (six to 36 months).

We present the case of a healthy 16-month-old infant who was brought to the Emergency Department with what appeared to be a “black hole” on the roof of her mouth. Subsequent evaluation revealed the object to be a decorative emblem dislodged from a car key fob, firmly adhered to the hard palate by residual adhesive. This report emphasizes the diagnostic pitfalls associated with radiolucent palatal foreign bodies and reinforces the essential role of careful history-taking, thorough oral examination, and early recognition in preventing unnecessary investigations and parental distress.

## Case presentation

**Figure 1:**
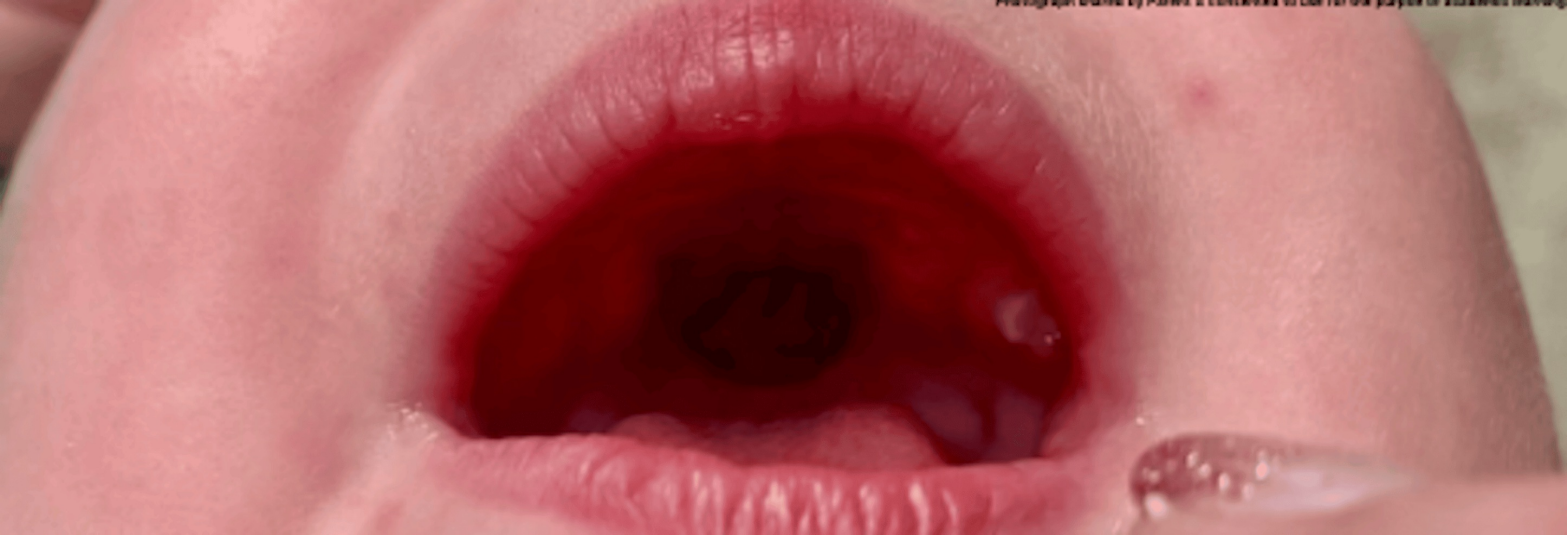
Intra oral examination finding with an apparent black hole

A 16-month-old infant was brought to the Emergency Department by her mother and grandmother, who noticed a sudden appearance of a “black growth” on the roof of her mouth (Figure 1).

The child was asymptomatic, feeding well, and meeting developmental milestones.

On examination, a black, slimy object was observed adhering to the hard palate. The lesion felt gritty to the touch, and initial attempts at dislodgement were unsuccessful. Radiographic imaging was inconclusive. Differential diagnoses included a button battery, a plastic foreign body, or early necrosis.

Closer inspection revealed a faint embossed symbol resembling the Volkswagen (VW) logo. Upon further inquiry, the father confirmed owning a VW vehicle and reported a missing emblem from his key fob. This led to the diagnosis: the infant had inadvertently affixed the key fob’s plastic logo sticker onto her palate, where the adhesive caused firm attachment.

**Figure 2:**
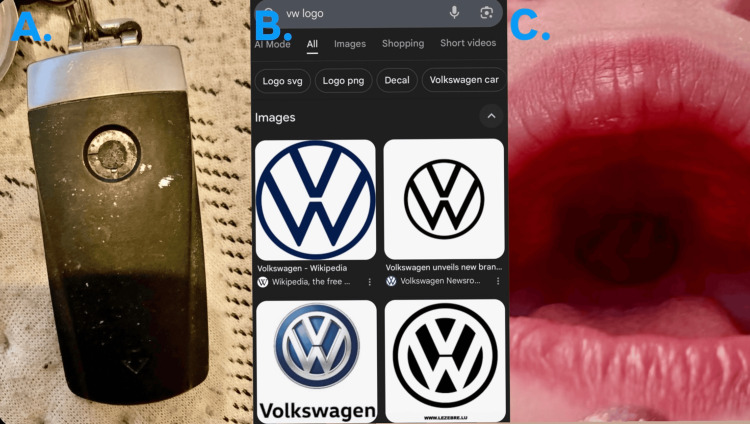
(A) Image with reference of the key fob, (B) VW reference logo, and (C) Label pin stuck to the palate inside the mouth of the patient

The patient was taken to the operating theatre, and under general anesthesia, the object was safely removed without aspiration or trauma. She was discharged the same day with an appropriate safety-netting advice (Figure 2).

## Discussion

Foreign bodies within the oral cavity of infants and toddlers represent a rare but clinically significant diagnostic challenge. Because this age group is prone to exploratory mouthing and insertion of small objects, clinicians often encounter unusual presentations that mimic more serious pathology [1,2]. The present case exemplifies how a simple, radiolucent object, in this instance, a plastic emblem from a car key fob, can produce a deceptive clinical picture that initially raised concern for mucosal necrosis or a retained button battery.

Mimicry of oral pathology

The potential for benign foreign bodies to mimic pathological oral lesions has been repeatedly noted in pediatric literature. Small, flat, or adhesive materials can appear as pigmented, ulcerated, or indurated plaques and are easily mistaken for vascular or neoplastic lesions. In some reports, these objects remained in situ for prolonged periods before diagnosis. For instance, Tewari et al. [4] described a case in which a plastic sticker adhered to the hard palate for four months before its true nature was recognized, having been initially misinterpreted as a salivary gland tumor. Similarly, other studies documented foreign bodies retained for more than a year, presenting as persistent palatal swellings [5,6]. These cases underscore that clinicians must maintain a high index of suspicion for foreign material whenever a new or unexplained intra-oral lesion appears in a child.

Diagnostic limitations and imaging challenges

The diagnostic process is often complicated by the fact that many such objects, including plastic, wood, or organic materials, are radiolucent and therefore invisible on plain radiographs. In our case, radiographic imaging was inconclusive, failing to distinguish the object from surrounding mucosa. This limitation mirrors prior reports, such as the account of Farhat et al. of an eight-month-old with a hard palatal “mass” that was misinterpreted radiologically as a possible tumor until surgical exploration revealed a pistachio shell [3]. These experiences demonstrate that the absence of radiographic evidence does not rule out a foreign body and should not delay further evaluation, particularly when the clinical history or examination raises suspicion.

Clinical subtlety and the importance of history

The absence of overt symptoms can also delay diagnosis. Our patient remained systemically well, afebrile, and continued to feed normally, consistent with several previous cases of retained palatal foreign bodies that elicited minimal inflammation. Nevertheless, certain materials, such as button batteries or chemically active adhesives, can cause mucosal necrosis within hours, necessitating urgent recognition and removal. The gritty surface and dark coloration in our case initially raised concern for a corrosive process, but the discovery of an embossed logo provided the crucial diagnostic clue. This finding highlights how detailed history-taking and caregiver questioning, specifically about household items, toys, or keys, can yield critical information that prevents misdiagnosis.

Management and safety considerations

Definitive management generally requires removal under general anesthesia, particularly in infants or uncooperative toddlers, to minimize aspiration risk and ensure atraumatic extraction [7]. In our patient, theatre removal was uneventful, and the palate healed without sequelae. Post-removal safety-netting and parental education regarding small detachable objects are essential components of care, as recurrent episodes may occur if environmental hazards persist.

Learning points and clinical implications

This case highlights several key learning points for emergency and pediatric clinicians: (1) Maintain suspicion for foreign bodies when assessing any unusual or pigmented lesion in the oral cavity of a child, particularly in the six- to 36-month age range; (2) Recognize imaging limitations, the absence of a radiographic finding does not exclude the presence of a foreign object; (3) Take an environmental history; parental input about missing toys, household items, or keychains can provide decisive diagnostic clues; (4) Ensure timely removal under controlled conditions to avoid aspiration or mucosal trauma; (5) Provide preventive education to caregivers about the hazards of small detachable objects and the importance of supervision during play.

In summary, our case adds to the small but growing literature describing palatal foreign bodies masquerading as oral lesions. It reinforces the necessity for vigilance, thorough examination, and strong clinical suspicion before pursuing invasive or imaging-heavy investigations. Adhering to these principles can minimize parental distress, reduce healthcare costs, and ensure safe outcomes for young patients presenting with deceptively simple findings.

## Conclusions

This case underscores the diagnostic complexity that can arise from seemingly benign findings in pediatric patients. A foreign body tightly adherent to the hard palate may closely mimic a pathological lesion, prompting unnecessary investigations and significant caregiver anxiety. Maintaining a high index of suspicion, particularly in infants and toddlers within the mouthing age range, is essential when assessing any atypical intra-oral discoloration or lesion.

Early and meticulous clinical examination, supported by a careful environmental history, remains the cornerstone of diagnosis. As this case demonstrates, the absence of radiographic evidence does not exclude the presence of a foreign body, especially when composed of plastic or adhesive materials. Timely recognition and controlled removal under appropriate anesthesia not only prevent complications such as aspiration or mucosal injury but also restore parental reassurance and avoid unnecessary use of hospital resources.

Ultimately, this report reinforces a simple but crucial lesson: in pediatrics, the most alarming presentations may occasionally conceal the most benign causes. Awareness of this possibility among emergency, pediatric, and dental practitioners can prevent misdiagnosis, reduce procedural risk, and promote safe, effective, and compassionate patient care.
